# Calcein+/PI- as an early apoptotic feature in *Leishmania*

**DOI:** 10.1371/journal.pone.0187756

**Published:** 2017-11-07

**Authors:** Louise Basmaciyan, Nadine Azas, Magali Casanova

**Affiliations:** Aix Marseille Univ, Univ Montpellier 1, IRBA, IP-TPT, Marseille, France; Instituto Oswaldo Cruz, BRAZIL

## Abstract

Although leishmaniases are responsible for high morbidity and mortality all over the world, no really satisfying treatment exists. Furthermore, the corresponding parasite *Leishmania* undergoes a very characteristic form of programmed cell death. Indeed, different stimuli can induce morphological and biochemical apoptotic-like features. However, the key proteins involved in mammal apoptosis, such as caspases and death receptors, are not encoded in the genome of this parasite. Currently, little is known about *Leishmania* apoptosis, notably owing to the lack of specific tools for programmed cell death analysis in these parasites. Furthermore, there is a need for a better understanding of *Leishmania* programmed cell death in order (i) to better understand the role of apoptosis in unicellular organisms, (ii) to better understand apoptosis in general through the study of an ancestral eukaryote, and (iii) to identify new therapeutic targets against leishmaniases. To advance understanding of apoptosis in *Leishmania*, in this study we developed a new tool based on the quantification of calcein and propidium iodide by flow cytometry. This double labeling can be employed to distinguish early apoptosis, late apoptosis and necrosis in *Leishmania* live cells with a very simple and rapid assay. This paper should, therefore, be of interest for people working on *Leishmania* and related parasites.

## Introduction

While apoptosis has been largely recognized in metazoans for several decades, programmed cell death (PCD) in unicellular organisms remains controversial [1]. Currently, PCD in unicellular organisms, and especially in the flagellated protozoan parasite *Leishmania*, has been accepted by most people, being described as a selfish altruism [2]. Indeed, parasite PCD may regulate parasite densities within the vector and within the mammalian host [3]. It may also modulate host immunity to allow successful infection [3]. In *Leishmania*, PCD has been reported in response to a wide range of stimuli, notably leishmanicidal drugs such as miltefosine [4], curcumin [5], or pentamidine [6] or the molecule H_2_O_2_ [7]. This PCD shares common phenotypical and biochemical features with mammalian apoptosis. These features include cell rounding up, reduction of cellular volume, plasma membrane modifications with maintenance of integrity, chromatin condensation, oligonucleosomal DNA fragmentation and mitochondrial depolarization [8]. According to the Nomenclature Committee on Cell Death, the term ‘apoptosis’ describes a specific morphological aspect of cell death and so should be applied to cell death events that occur while manifesting several apoptotic morphological features [9]. Among these features, we can cite, *in vitro*, cell rounding-up, retraction of pseudopodes, reduction of cellular volume, chromatin condensation, nuclear fragmentation, little or no ultrastructural modifications of cytoplasmic organelles, plasma membrane blebbing with maintenance of its integrity [9]. As a consequence, we can talk about not only PCD in *Leishmania* but also apoptosis. For the moment, the cell death pathways involved and the executioner proteins remain largely unknown. Indeed, neither death receptors, nor caspases, which are key proteins of mammalian apoptosis, are present in the genome of *Leishmania* [1]. It is only recently that a member of the Bcl-2 family of proteins has been identified in *L*. *infantum* [10]. The apoptotic pathways are still largely unknown in *Leishmania* notably due to the lack of specific apoptotic markers. Indeed, morphological changes with cell rounding-up and cell shrinkage appear during parasite apoptosis, as well as a mitochondrial depolarization [8]. However, these features are not specific for apoptosis and could appear under other stress conditions. Propidium iodide (PI) is indicative of cell death but is not specific to apoptosis. Indeed, PI enters the cell only if the membrane has lost its integrity, *i*.*e*. during late apoptosis or necrosis, but not during early apoptosis (reviewed in Atale *et al*.[11]). Furthermore, *Leishmania* cells lack any detectable levels of phosphatidylserine, a phospholipid that is exposed at the surface of metazoan cells in response to apoptotic stimuli [12]. Since Annexin V binds other phospholipid classes in *Leishmania* [12], its staining is indicative of membrane modifications and is not an apoptosis marker in this parasite. Caspase-like activity assays are also used by authors to prove that *Leishmania* cells are in apoptosis [8]. However, no conclusions can be drawn from these experiments until the enzyme(s) involved has (have) been molecularly characterized. Last, important nuclear changes appear during apoptosis, including (i) morphological changes which are poorly described in *Leishmania* apoptosis and are not specific to apoptosis, (ii) chromatin condensation which is difficult to highlight in *Leishmania* [8], and (iii) DNA fragmentation. To detect DNA degradation, a TUNEL assay has been developed, based on fluorochrome-labeling of 3’-OH termini of DNA strand breaks *in situ* with the use of exogenous terminal deoxynucleotidyl transferase TdT. However, as the technique requires a preliminary cell fixation to allow the enzyme to enter the cells, it cannot be used on live cells. Furthermore, since nuclear staining corresponds to DNA degradation and not to background staining, fluorescence microscopy is recommended, which is time-consuming.

In mammal cells, it has been demonstrated that the use of acetoxymethyl ester of calcein (calcein-AM) in association with ethidium homodimer allows the evaluation of cell apoptosis through detection of esterase activity and cellular membrane physical and chemical alterations in adherent [13] and non-adherent cells [14]. The authors also demonstrated that beside the clear definition of viable, apoptotic and necrotic dead cell populations, calcein-AM/Ethidium homodimer labeling was more sensitive for apoptotic cell quantification than Annexin V/PI [12,13].

In this study, we developed a novel very simple and quick tool for studying apoptosis in *Leishmania* using flow cytometry, which can be performed on live cells. This tool, based on calcein/PI double labeling, makes it possible to discriminate between early apoptosis, late apoptosis and necrosis in *Leishmania* cells.

## Materials and methods

### Cells

*L*. *major* wild-type parasites MRHO/IR/75 promastigotes from the CNR *Leishmania* (Montpellier, France) were grown in Schneider’s *Drosophila* medium supplemented with 100U/mL penicillin, 100μg/mL streptomycin, 1% L-glutamine 200mM and 20% heat inactivated fetal calf serum (FCS) (Gibco^®^, Life Technologies, France) at 26°C.

The THP-1 cell line (acute monocytic leukemia cell line purchased from ATCC, ref TIB-202) was cultivated in RPMI medium supplemented with 100U/mL penicillin, 100μg/mL streptomycin, 1% L-glutamine 200mM and 10% FCS at 37°C (Gibco^®^, Life Technologies, France) with 5% CO_2_.

### Induction of cell death

Cell death was induced by harvesting logarithmic *L*. *major* cells by centrifugation at 600g for 10min and incubating cells at 10^7^cells/mL in culture medium at 26°C with different concentrations of drugs. Miltefosine was sourced from Santa Cruz Biotechnology (USA) while other drugs came from Sigma-Aldrich Inc. (USA).

### Calcein/PI labeling

For flow cytometry, cells were washed once in PBS and resuspended in 1mL of calcein (LIVE/DEAD^®^ Viability/Cytotoxicity Kit for mammalian cells, Molecular Probes, OR, USA) diluted 1/80 in DMSO and 5μL Propidium Iodide (PI) at 0.5mg/mL. The mixed sample was then incubated for 15–20 minutes at room temperature, protected from light. The cells were analyzed by flow cytometry using 488nm excitation and measuring green fluorescence emission for calcein (530/30 bandpass) and red fluorescence emission for PI (610/20 bandpass) on the BD LSRFortessa^™^ cell analyzer (BD Biosciences, France). Data were exported and analyzed with the Flowjo software.

For fluorescence microscopy, harvested cells were fixed in 4% paraformaldehyde (PFA) at 4°C for 30min. After a PBS wash, cells were adhered to a microscope fluorescence slide. The slides were mounted with SlowFade Gold antifade mountant with DAPI (Life Technologies, Saint-Aubin, France). Observations were carried out using a BX51 fluorescence microscope (Olympus, Rungis, France) and images acquired using the fluorescence imaging system Cell^A^ (Olympus, Rungis, France).

### TUNEL

To detect DNA double-strand breaks, we applied the TUNEL test using the *in situ* cell death detection kit, fluorescein (Roche, Meyla, France). Cells were fixed with paraformaldehyde 4%, laid on an immunoslide and permeabilized with a 0.1% triton X-100 and 0.1% sodium citrate solution for 2min at 4°C. After three washes in PBS, the enzyme was added, diluted 1/10 in the kit buffer. The cells were washed five times in PBS before air drying and the addition of SlowFade Gold antifade mountant with DAPI (Life Technologies, Saint-Aubin, France). Cells were observed using a BX51 fluorescence microscope (Olympus, France). Bright field and fluorescence images were acquired using the fluorescence imaging system Cell^A^ (Olympus, Rungis, France).

### Statistical analysis

For statistics, unpaired t-tests or an ANOVA test were performed (BioStaTGV). Results were considered statistically significant when p<0.05. For significant differences, * means p<0.05, ** p<0.01 and *** p<0.001. For the correlation test, a Pearson test was carried out using the Rweb site (https://rweb.stat.umn.edu/Rweb/Rweb.general.html). The number of experiments performed (minimum 3) is detailed in each Figure (n).

## Results

### Calcein labels viable mammalian cells but not *Leishmania* viable cells

**Fig 1:**
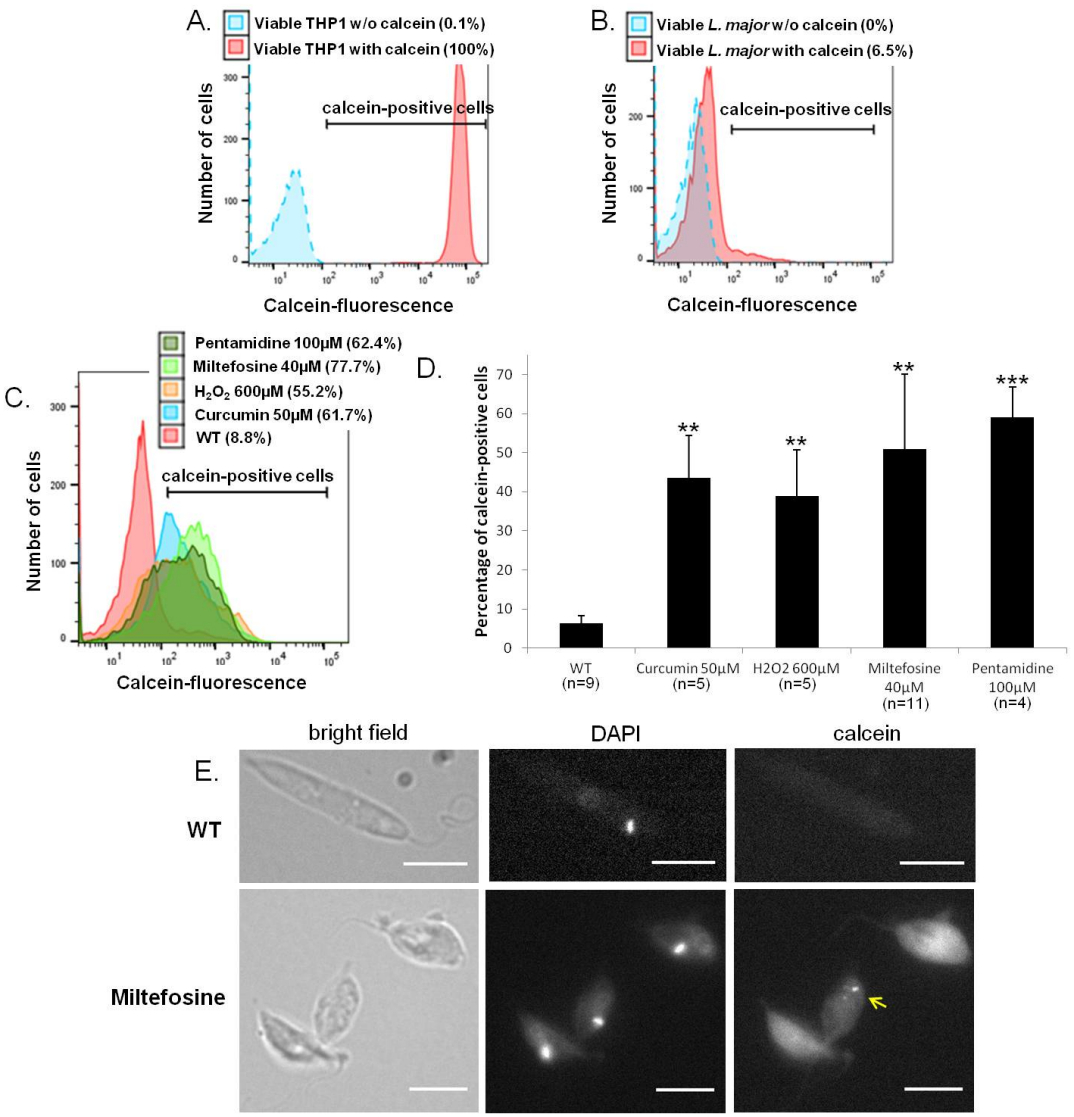
Calcein labels mammalian viable cells and dead but not viable *Leishmania* cells. (A-B) Raw histograms from flow cytometer analysis of viable THP1 cells (A) or viable *L*. *major* cells (B) stained or not stained with calcein-AM. The percentage of calcein-positive cells is given in brackets in the legend. All viable THP1 cells appeared stained with calcein-AM, while viable *L*. *major* cells were not stained. The same result has been obtained in at least four independent experiments. (C) Raw histograms from flow cytometer analysis of viable *L*. *major* cells (WT) and of apoptotic cells stained with calcein-AM. Cell apoptosis was induced for 24h either with 50μM of curcumin, 600μM of H_2_O_2_, 40μM of miltefosine, or 100μM of pentamidine. (D) Mean percentage (±SD) of calcein-positive cells in viable WT cells and after the addition of four pro-apoptotic drugs for 24h. The number of experiments performed (n) is given for each condition. Unpaired t-test, ** p<0.01 and *** p<0.001. (E) Microscopical observation of WT *L*. *major* cells (upper panels) and *L*. *major* cells treated with 40μM of miltefosine for 24h (lower panels) and labeled with calcein-AM. Calcein gathered in some dead cells in punctuated cytoplasmic structures (arrow) (bar = 5μm).

Calcein-AM is a virtually non-fluorescent cell-permeant neutral dye that is converted by cell esterases into its negative impermeant green fluorescent analogue [15]. This dye is well retained within viable mammalian cells, producing an intense green fluorescence in 100% of the THP1 cells as can be seen in Fig 1A. In contrast, *L*. *major* viable cells were not labeled with calcein (Fig 1B).

### Calcein labels *Leishmania* dead cells

We then tested calcein-AM on *L*. *major* dead cells. To induce cell death, we used miltefosine, a pro-apoptotic drug in *Leishmania* [4]. Indeed, this drug has a cytotoxic effect on *Leishmania* cells *in vitro* and different features of apoptosis were observed when *Leishmania* cells were cultivated with it: cell shrinkage, cell rounding up and DNA fragmentation while maintenance of plasma membrane integrity [4]. As shown in Fig 1C and 1D, a high *L*. *major* labeling was observed after the addition of 40μM of miltefosine for 24h. On average, 51% of miltefosine-incubated cells were calcein-positive, while only 11% were labeled without the apoptotic drug (Fig 1C).

To ensure that this effect was not linked to the miltefosine drug but was due to cell death independently of the stimulus, we induced *L*. *major* death with three other molecules described as pro-apoptotic in *Leishmania*: curcumin, H_2_O_2_ and pentamidine [5–7]. It can be seen in Fig 1C and 1D that calcein-AM labeled dead cells independently of the drug added with minimum 40% of calcein-positive cells on average. Using fluorescence microscopy, we observed that calcein gathered in some cells in punctuated cytoplasmic structures (Fig 1E), certainly calcium-rich regions of the cytoplasm since calcein is a calcium-dependent fluorescent molecule.

### Calcein/PI labeling allows distinguishing early apoptosis from late apoptosis and necrosis

**Fig 2:**
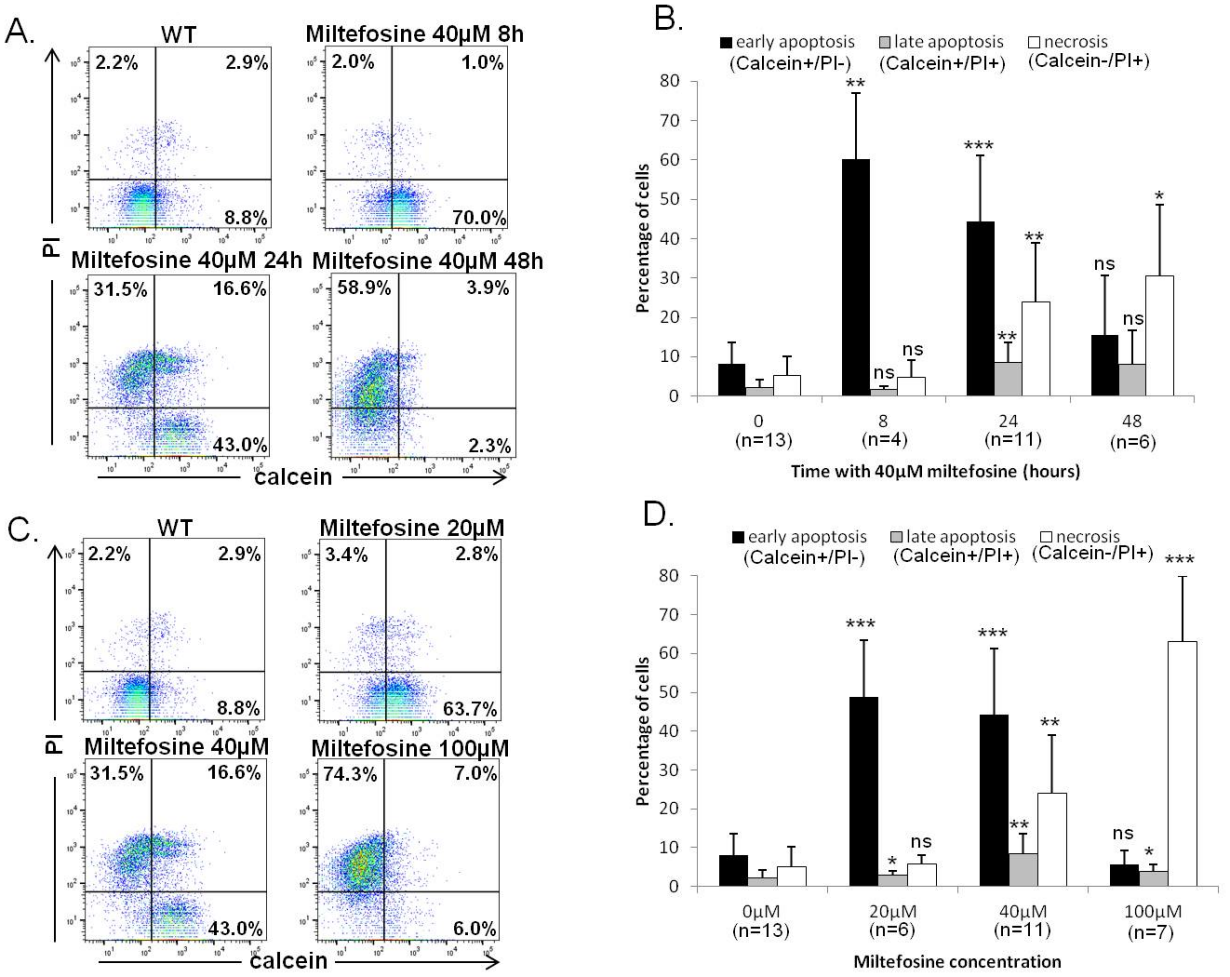
Calcein/PI labeling makes it possible to distinguish early apoptosis from late apoptosis and necrosis. (A-B) Quantification of calcein and PI staining in WT cells and in cells incubated with 40μM of miltefosine for 8h, 24h and 48h: raw data for one experiment representative of at least four experiments (A) and mean percentage (±SD) of cells in early apoptosis (calcein+/PI-), late apoptosis (calcein+/PI+) and necrosis (calcein-/PI+). Unpaired t-test, * p<0.05, ** p<0.01 and *** p<0.001. (C-D) Quantification of calcein and PI staining in WT cells and in cells incubated with increasing concentrations of miltefosine: 20μM, 40μM or 100μM for 24h: raw data for one experiment representative of at least six experiments (C) and mean percentage of cells ±SD (D). Unpaired t-test, * p<0.05, ** p<0.01 and *** p<0.001.

When we carried out calcein/propidium iodide (PI) double labeling and followed cells at different times after adding 40μM of miltefosine, changes in the calcein/PI labeling were observed. Indeed, the cell population switched from no labeling (calcein-/PI-) to calcein labeling without any PI labeling (calcein+/PI-) after eight hours, then to a double calcein/PI labeling (calcein+/PI+) and, finally, to PI labeling without any calcein labeling (calcein-/PI+) (Fig 2A). However, PI enters cells characterized by membrane degradation, a feature of late apoptosis, and cells without any membrane, a feature of necrosis (reviewed in Atale *et al*.[11]). As a consequence, the calcein+/PI- population represents cells into which calcein can enter, *i*.*e*. cells with membrane modifications but with membrane integrity (PI-negative), the feature of cells in early apoptosis (reviewed in Atale *et al*. [11]). The calcein+/PI+ population corresponds to cells in late apoptosis with a degraded membrane. The calcein-/PI+ population corresponds to cells in necrosis (membrane loss) (reviewed in Atale *et al*.[10]).

We quantified cells in early or late apoptosis and in necrosis according to the incubation time with 40μM of miltefosine, using calcein/PI double labeling. We observed that eight hours of incubation with 40μM of miltefosine induced a significant increase in the percentage of early apoptotic cells, whereas incubation for 24h induced the appearance of cells in late apoptosis and in necrosis (Fig 2B). Incubation for 48h with 40μM of miltefosine induced a significant increase in the percentage of necrotic cells (Fig 2B).

These results were confirmed by carrying out calcein/PI double labeling after incubation for 24h with different miltefosine concentrations. As previously shown, we observed a switch in the population from calcein-/PI- (WT viable cells) to calcein+/PI- (with 20μM of miltefosine), to calcein+/PI+ (with 40μM of miltefosine) and then to calcein-/PI+ (with 100μM of miltefosine) (Fig 2C). Accordingly, when quantifying the apoptotic cells, we observed a significant proportion of cells in early apoptosis with 20μM of miltefosine, in late apoptosis with 40μM of miltefosine and in necrosis with 100μM of miltefosine (Fig 2D). Thus, our results demonstrated that the calcein/PI double labeling makes it possible to distinguish early apoptosis from late apoptosis and necrosis.

### Calcein+/PI- is a very early apoptotic feature of *Leishmania* cells

**Fig 3:**
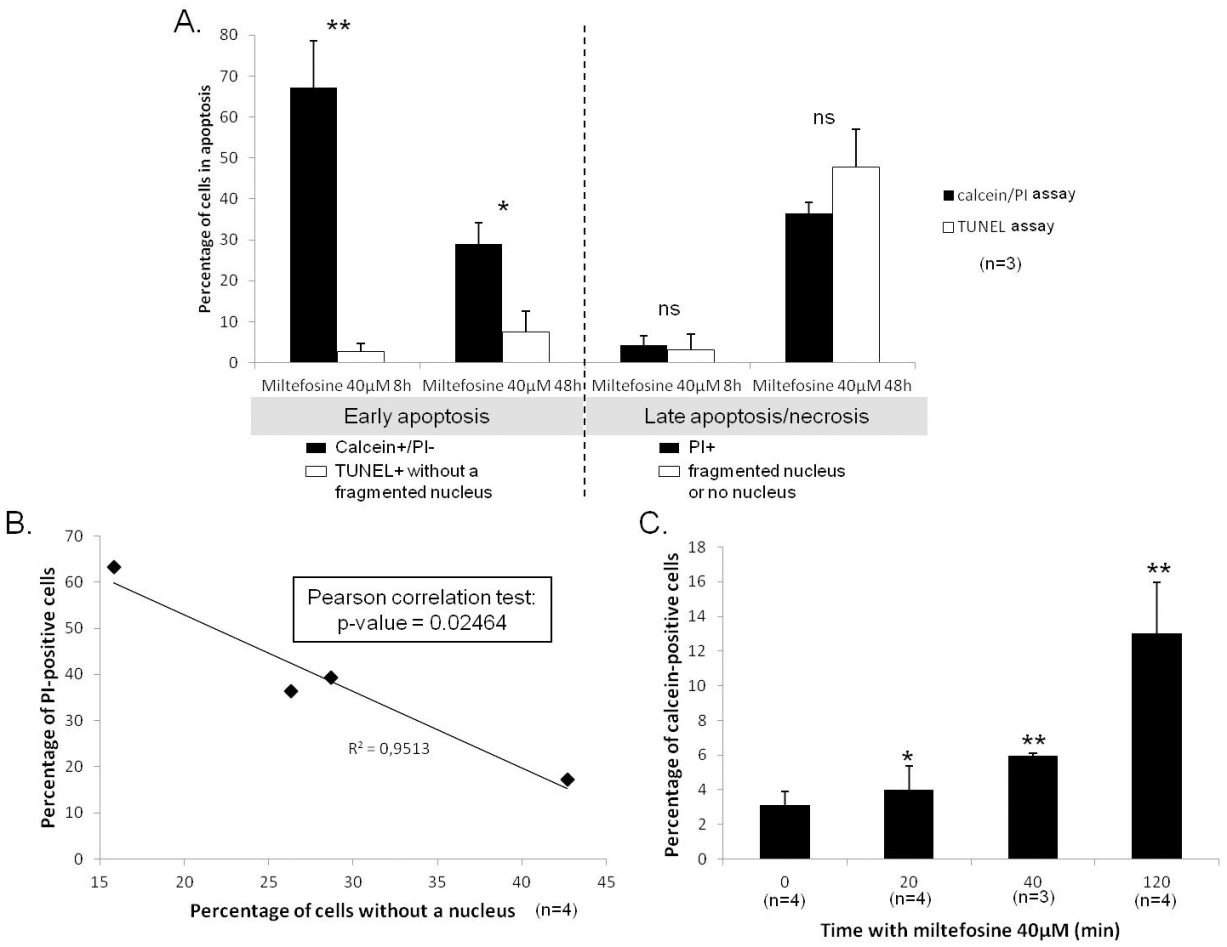
Calcein+/PI- is a very early apoptotic feature of *Leishmania* cells. (A) Percentage of cells in early apoptosis or in late apoptosis/necrosis, according to a calcein/PI labeling or to a TUNEL assay: mean ±SD. Cells were considered to be in early apoptosis when they were calcein+/PI- according to the calcein/PI labeling and TUNEL-positive with a non-degraded nucleus according to the TUNEL assay. For late apoptosis/necrosis, PI-positive cells (calcein/PI labeling) and cells with a degraded or lost nucleus (TUNEL assay) were considered. Significant differences appeared between both techniques concerning the percentage of early apoptotic cells, whereas the percentages of late apoptotic/necrotic cells were the same with the two assays, according to three independent experiments. Unpaired t-test, * p<0.05 and ** p<0.01. (B) Correlation between the percentage of PI-positive cells and of cells without a nucleus. (C) Percentage of calcein-positive cells after incubation of the cells for 20min, 40min or 120min with 40μM of miltefosine: mean ±SD. Unpaired t-test, * p<0.05 and ** p<0.01.

As mentioned in the introduction, currently, the only available tool to demonstrate apoptosis in *Leishmania* cells and to discriminate between early and late apoptosis is the TUNEL assay. Hence, we decided to compare the calcein/PI and the TUNEL assays for the description of apoptosis. To do so, we used both methods to quantify cells in early apoptosis (calcein+/PI- cells or TUNEL+ cells with a non-degraded nucleus) and cells in late apoptosis/necrosis (PI+ cells or cells with a degraded or lost nucleus). We noted that the percentage of cells in late apoptosis/necrosis was similar in both assays, no significant difference being observed (Fig 3A). On the contrary, significant differences appeared in the percentage of early apoptotic cells depending on the assay used. Indeed, while almost no TUNEL-positive cells could be observed after 8h with 40μM of miltefosine, almost 70% of cells in early apoptosis were detected with the calcein/PI double labeling (Fig 3A). We can conclude that calcein/PI is able to detect apoptosis earlier than the TUNEL assay.

We noted that PI permeabilization during late apoptosis/necrosis could be underscored as a consequence of DNA degradation. Indeed, cells became PI-negative shortly after cell death as a consequence of a reduction in the amount of DNA inside the cells, as shown by the correlation between the percentage of PI-positive cells and the percentage of cells without a nucleus in Fig 3B (p-value Pearson correlation test: 0.02464).

In order to show that calcein is a very early apoptotic marker, we quantified the percentage of calcein-positive cells using flow cytometry after only 20, 40 and 120min with 40μM of miltefosine. Fig 3C shows a significant increase in the percentage of calcein-positive cells, even after only 20min with miltefosine, a condition under which no cell concentration decrease could be observed. Calcein is, therefore, a very early apoptotic marker in *Leishmania*.

## Discussion

**Fig 4:**
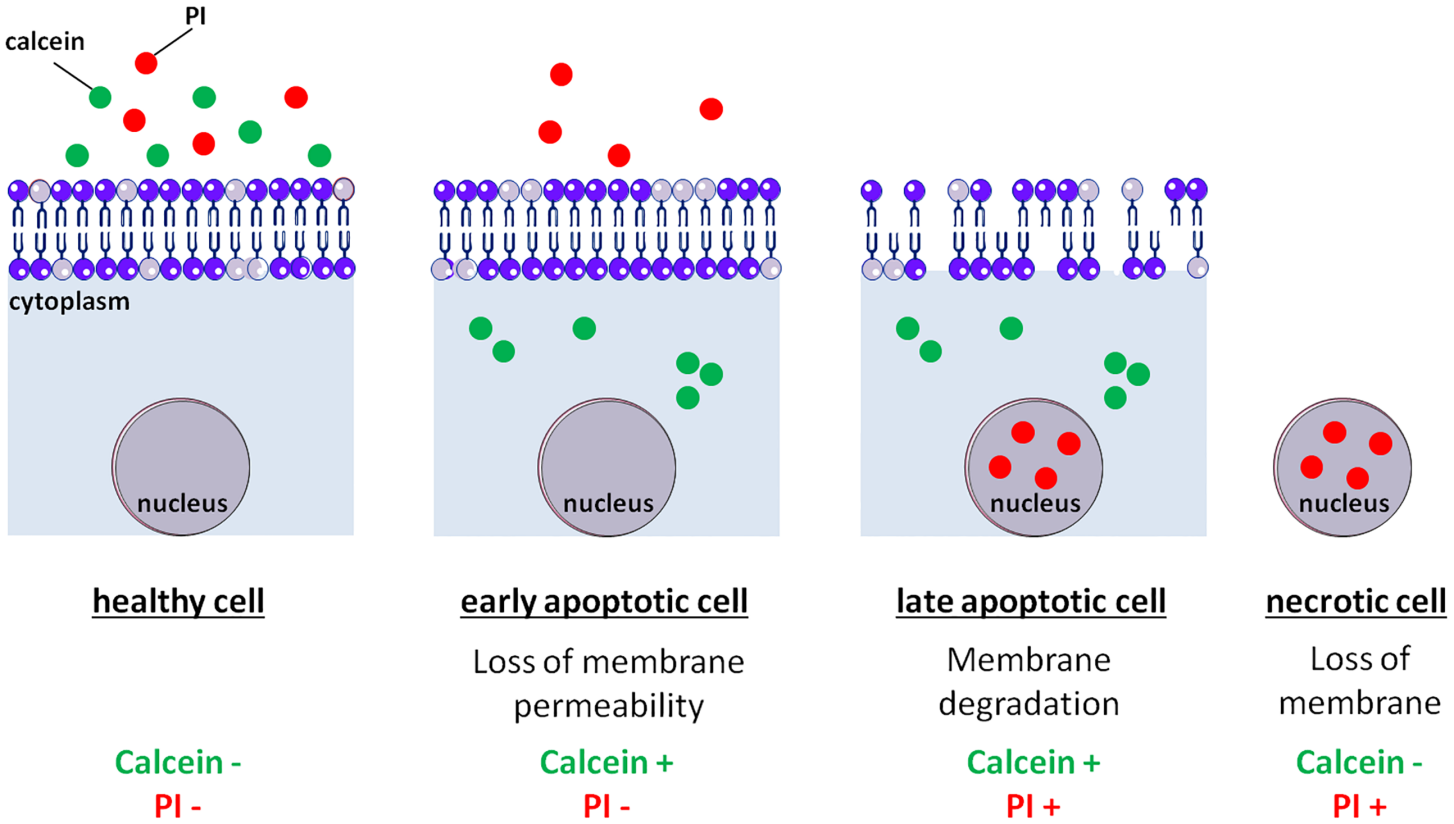
Model: calcein/PI labeling in *Leishmania*. In viable cells, the membrane properties prevent calcein and PI from entering the cells which appear calcein-/PI-. During early apoptosis, very early membrane modifications induce a high plasma membrane permeability, inducing calcein entry. Membrane integrity maintenance prevents PI from entering the cells. As a consequence, cells in early apoptosis appear calcein+/PI-. During late apoptosis, membrane degradation creates pores in the plasma membrane allowing PI to enter the cells which are calcein+/PI+. Finally, *in vitro*, in the absence of phagocytosis, cells enter necrosis characterized by membrane loss, so these last cells appear calcein-/PI+.

In this study, we identified that *Leishmania* cells labeling with calcein and PI can be employed to distinguish the different phases of apoptosis: early apoptosis, late apoptosis and necrosis, as presented in Fig 4. Indeed, calcein cannot enter *Leishmania* viable cells, contrary to mammal viable cells. The composition of the parasite membrane, which is different to that of mammal cells, could explain this difference. For example, mammal cholesterol is replaced by ergosterol in the parasite membrane [16]. This could also been explained by the presence of pumps that actively export calcein as described by some authors [17]. When the cell enters apoptosis, calcein enters the cell. This could be explained by membrane modifications during apoptosis, inducing higher plasma membrane permeability. The export pumps could also be inhibited during early apoptosis, preventing calcein extrusion from the parasite. In the cytoplasm, calcein is converted by esterases into a fluorescent molecule trapped in the cytoplasm [13]. It was noted that the high calcium concentration in *Leishmania* apoptotic cells [18] certainly induces a high accumulation of calcein in the cytoplasm of apoptotic cells, since calcein fluorescence is calcium-dependent. The formation of calcein aggregates could be due to calcium-rich microdomains in the cytoplasm (reviewed in [19]). On the other hand, the maintenance of membrane integrity prevents PI entry. As a consequence, cells in early apoptosis appear calcein+/PI- (Fig 4). The plasma membrane then deteriorates, a feature of late apoptosis [11]. As a consequence, PI enters into the cells, while calcein is still trapped: the cells are calcein+/PI+ (Fig 4). Finally, the plasma membrane is lost *in vitro* in the absence of phagocytosis [9] and calcein is released. Hence, the cells appear calcein-/PI+ (Fig 4). As a consequence, calcein/PI double labeling makes it possible to distinguish between early apoptosis, late apoptosis and necrosis. It is important to note that in order to ensure that a stimulus induces apoptosis, a switch of the population from healthiness (calcein-/PI-) to early apoptosis (calcein+/PI-) and then to late apoptosis or necrosis (PI+) must be observed.

It should be noted that the use of calcein/PI as an apoptotic marker in *Leishmania* has been demonstrated independently of the stimulus, since we reached the same result with four different pro-apoptotic molecules. An ANOVA test even confirmed the absence of differences in the percentage of calcein-positive cells with the four different drugs (p = 0.22). This confirms the possibility of using this assay to define *Leishmania* apoptosis induced by different drugs.

In *Leishmania* cells, very few markers can be used to characterize apoptosis, notably owing to the lack of phosphatidylserine [12]. The only specific marker for identifying apoptosis is, therefore, DNA fragmentation while maintaining plasma membrane integrity [8]. However, DNA fragmentation appears relatively late during apoptosis [20], largely after the first membrane modifications characterizing early apoptosis. Furthermore, since several difficulties are encountered when analyzing it in agarose gels in protozoans [8], DNA fragmentation is usually highlighted by a TUNEL assay, which requires previous cell fixation to enable the enzyme to reach the DNA. In this study, we have shown that calcein+/PI- labeling is detected earlier than DNA fragmentation and can be employed as a feature of early apoptosis. Furthermore, the calcein/PI assay is relatively fast, requiring no previous cell fixation, and is less tedious than the TUNEL assay since the quantification of apoptotic cells appears immediately (there is no need to analyze fluorescence images).

## Conclusion

In conclusion, in this study we identified a new apoptotic marker in the protozoan parasite *Leishmania*, where the absence of tools for studying apoptosis resulted in a lack of knowledge about the phenomenon. This new tool will contribute to a better understanding of apoptosis in *Leishmania* and so of the role of this process in unicellular organisms. It will also contribute to a better understanding of apoptosis in eukaryotes in general, through the study of an ancestral eukaryote. Finally, it will enable the identification of new therapeutic targets against a parasitic disease which is responsible for high morbidity and mortality all over the world and for which no satisfying treatment exists.
